# AAp-MSMD: Amino Acid Preference Mapping on Protein–Protein Interaction Surfaces Using Mixed-Solvent Molecular Dynamics

**DOI:** 10.1021/acs.jcim.3c01677

**Published:** 2023-12-12

**Authors:** Genki Kudo, Keisuke Yanagisawa, Ryunosuke Yoshino, Takatsugu Hirokawa

**Affiliations:** † Physics Department, Graduate School of Pure and Applied Sciences, University of Tsukuba, 1-1-1 Tennodai, Tsukuba 305-8571, Ibaraki Japan; ‡ Department of Computer Science, School of Computing, Tokyo Institute of Technology, 2-12-1 Ookayama, Meguro 152-8550, Tokyo Japan; § Middle Molecule IT-based Drug Discovery Laboratory, Tokyo Institute of Technology, 2-12-1 Ookayama, Meguro 152-8550, Tokyo Japan; ∥ Faculty of Medicine, University of Tsukuba, 1-1-1 Tennodai, Tsukuba 305-8575, Ibaraki Japan; ⊥ Transborder Medical Research Center, University of Tsukuba, 1-1-1 Tennodai, Tsukuba 305-8577, Ibaraki Japan

## Abstract

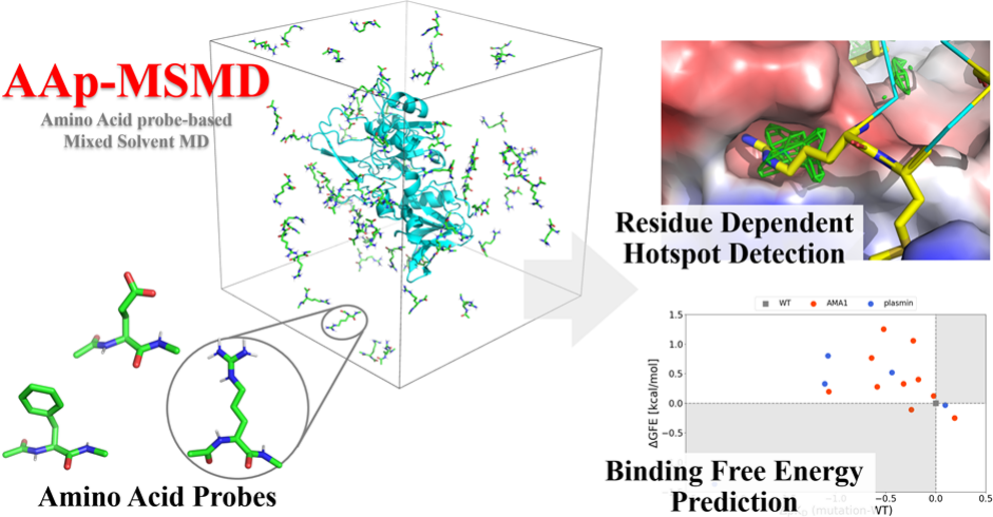

Peptides have attracted much attention recently owing to their well-balanced properties as drugs against protein–protein interaction (PPI) surfaces. Molecular simulation-based predictions of binding sites and amino acid residues with high affinity to PPI surfaces are expected to accelerate the design of peptide drugs. Mixed-solvent molecular dynamics (MSMD), which adds probe molecules or fragments of functional groups as solutes to the hydration model, detects the binding hotspots and cryptic sites induced by small molecules. The detection results vary depending on the type of probe molecule; thus, they provide important information for drug design. For rational peptide drug design using MSMD, we proposed MSMD with amino acid residue probes, named amino acid probe-based MSMD (AAp-MSMD), to detect hotspots and identify favorable amino acid types on protein surfaces to which peptide drugs bind. We assessed our method in terms of hotspot detection at the amino acid probe level and binding free energy prediction with amino acid probes at the PPI site for the complex structure that formed the PPI. In hotspot detection, the max-spatial probability distribution map (max-PMAP) obtained from AAp-MSMD detected the PPI site, to which each type of amino acid can bind favorably. In the binding free energy prediction using amino acid probes, ΔGFE obtained from AAp-MSMD roughly estimated the experimental binding affinities from the structure–activity relationship. AAp-MSMD, with amino acid probes, provides estimated binding sites and favorable amino acid types at the PPI site of a target protein.

## Introduction

Drug modalities are diverse and can be classified into three types based on their molecular weights and characteristics: small molecules, macromolecules, and cyclic peptides. Small molecules with an approximate molecular weight of 500 Da are the most common modality in approved drugs.^1^ This modality can be administered orally and can permeate membranes.^2^ Additionally, small molecules can be chemically synthesized for industrial mass production. Although these advantages give it the largest market share, small molecules often present nonspecific interactions with deep hydrophobic pockets, making it difficult to ensure the selectivity of analogous proteins.^3^ Furthermore, macromolecules such as antibodies have high target selectivity and are expected to have fewer side effects.^2^ However, because of their molecular weight of 5–150 kDa,^4,5^ macromolecules are limited to extracellular targets without membrane permeability.^6^ Moreover, unlike small molecules, macromolecules cannot be chemically synthesized or administered orally.

Recently, cyclic peptides have attracted increasing attention as an alternative to small molecules and macromolecules. This modality is constructed from 4–15 amino acid residues and has a molecular weight of approximately 500–2000 Da.^2^ A typical cyclic peptide is cyclosporin, which is an immunosuppressant composed of 11 amino acid residues.^7^ Cyclic peptides, like small molecules, have superior membrane permeability, can be easily synthesized, and can be administered orally.^8−10^ Additionally, similar to macromolecules, cyclic peptides have high target selectivity and can inhibit protein–protein interactions (PPIs).^10,11^ Cyclic peptides are designed using optical isomers of natural amino acids, non-natural amino acids, and main-chain modification residues. Therefore, the number of combinations is not limited to 20 natural amino acids. Phage display screening and biopanning are typical methods for selecting optimal amino acid residues from a variety of combinations.^12,13^ While these screening methods are maturing, computational techniques are expected to identify peptide drug binding sites in target proteins and predict affinitive amino acid residues.^14^ Goldbach et al.^15^ used RaPID screening and docking simulation to predict protein–peptide complex structures with unknown binding mechanisms, demonstrating the importance of computational prediction.^16^ Understanding the inhibitory surfaces of PPIs is essential, considering the unclear mechanisms of action of cyclosporine, vancomycin,^17^ and gramicidin,^18^ which are cyclic peptides used in clinical practice.^19^

In drug discovery and lead optimization, computational methods, such as molecular simulation, are highly effective techniques and have been widely applied. MCSS and FTMap have been reported as hotspot search methods for protein surfaces to which small probe molecules bind.^20,21^ Although these methods can be applied to identify target sites and design PPI-inhibiting cyclic peptides, they do not consider competition with explicit water molecules and protein flexibility when searching for conformations because rigid body docking is employed.^21^ Molecular dynamics (MD) simulation is a method for predicting the time evolution of molecular physical movements based on Newton’s equations of motion and has been applied for biomolecules, such as proteins, nucleic acids, and lipid membranes.^22−24^ Additionally, MD is typically applied in hydration models filled with explicit water molecules. Mixed-solvent MD (MSMD) adds probe molecules or fragments of functional groups to the hydration model. Since MSMD considers the protein’s flexibility and can detect hotspots and cryptic sites where the probe can bind,^25−28^ it can be applied to predict the binding pocket appropriate for virtual screening.^29,30^ In addition, MSMD-detected binding hotspots are considered surfaces with a high affinity for probe molecules. Arcon et al. improved the performance of virtual screening by applying binding hotspot probes to perform small molecule pharmacophore matching and binding pose estimation.^31^ These results highlight the feasibility of MSMD-derived pharmacophore technology and demonstrate that identifying molecular structures with an affinity for protein surfaces is useful for drug design. Although these studies employed functional groups of small molecules (e.g., phenyl group) as probes, protein surfaces with a high affinity for amino acid residues can be predicted by performing MSMD using amino acid residue probes, considering protein flexibility and competition with water molecules.

In this study, we proposed an MSMD method using amino acid residues as probes, named amino acid probe-based MSMD (AAp-MSMD), to detect hotspot protein surfaces to which peptide drugs bind and to identify favorable amino acid residues. Notably, the difference between amino acids is in the side chain atoms; therefore, the hotspot detection procedure focuses on these atoms. Furthermore, to quantitatively predict the affinity of amino acid residues, we evaluated the binding free energy using grid free energy (GFE) and compared it with the reported structure–activity relationship (SAR) of peptide inhibitors.

## Materials and Methods

Our procedure was divided into three steps: MSMD simulation with amino acid probes, spatial probability distribution map (PMAP) generation specialized for amino acid probes, and GFE calculations.

### Preparation of Amino Acid Probes

First, the amino acid probes of interest were preprocessed. Each natural l-amino acid structure was downloaded from the RCSB Protein Data Bank (PDB),^32^ and the neutral main-chain terminus was modeled by adding *N*-methyl or acetyl groups. Next, the restrained electrostatic potential procedure (RESP) in the antechamber module of AmberTools21^33^ was employed to fit/convert the partial charges to reproduce the electrostatic potential, which was calculated using Gaussian 16 Rev B.01.^34^ First, all of the probe structures were optimized at the B3LYP/6-31G(d) level. Afterward, the electrostatic potentials were calculated at the HF level using the optimized structures. The centers of the electrostatic potentials were located at the center of each atom. The additional force field parameters for the probes were derived using the General AMBER Force Field 2 (GAFF2) rather than the Amber ff14SB force field because the amino acid probe was treated as a cosolvent monomer rather than as a residue on a protein.

### Mixed-Solvent Molecular Dynamics

After probe preparation, MSMD was performed following the protocol referring to EXPRORER.^35^ Notably, the initial positions of the probes affect the results, particularly in short MD simulations, and this initial position dependence influences the convergence of the analysis results. Therefore, the following protocols were independently performed 40 times with different initial probe coordinates to achieve efficient sampling. The procedure was divided into three steps, as described below.

### Initial System Generation

First, the probes were randomly placed around the protein at a concentration of 0.25 M using PACKMOL 18.169.^36^ A high concentration enables effective sampling of residue environments. Second, the system was solvated with water by using the LEaP module of AmberTools18. Na^+^ and Cl^–^ ions were added to neutralize the whole system. The Amber ff14SB force field and TIP3P model^37^ were used for protein and water molecules, respectively. Additionally, a pseudo Lennard-Jones force field term with the parameters (ϵ = 10^–6^ kcal/mol; *R*_min_ = 20 Å) was introduced between the center of the probes to prevent their aggregation.

### Minimization, Heating, and Equilibration

After constructing the initial structures, the systems were minimized to include 200 steps using the steepest descent algorithm with harmonic position restraints on the heavy solute atoms (force constant, 10 kcal/mol/Å^2^), and the systems were further minimized to 200 steps using the steepest descent algorithm without any position restraints. After minimization, the system was heated gradually to 300 K during 200 ps constant-NVT MD simulations with harmonic position restraints on the heavy solute atoms (force constant, 10 kcal/mol/Å^2^). During the subsequent 800 ps constant-NPT MD simulations at 300 K and 1 bar, the force constants of the positional restraints were gradually reduced to 0 kcal/mol/Å^2^. The P-LINCS algorithm^38^ was used to constrain all bond lengths involving hydrogen atoms, which allowed the use of 2 fs time steps. The temperature and pressure were controlled using a stochastic velocity rescaling algorithm,^39−41^ with a time constant of τ = 0.1 ps, and a Berendsen barostat,^42^ with a compressibility of 4.5 × 10^–5^ bar^–1^, respectively. Note that temperature was controlled in both the water group and nonwater group. Long-range Coulomb interactions were addressed by using the particle mesh Ewald summation method (PME), with the mesh spacing set to 1.2 Å and the nonbonded cutoff distance set to 10 Å. The simulations were performed using GROMACS 2021.5.^43^ The ParmEd module^44^ was used to convert the AMBER parameter/topology file format to the format used by GROMACS.

### Production Run

After equilibration, 40 ns constant-NPT MD simulations were performed at 300 K and 10^5^ Pa without position restraints. All settings were the same as those in the initial equilibration step, except that a Parrinello–Rahman barostat^45^ with a compressibility of 4.5 × 10^–5^ bar^–1^ was used instead of a Berendsen barostat. Snapshots were taken every 10 ps in the 20–40 ns range; therefore, 2000 snapshots were produced per MSMD simulation.

### Hotspot Detection with Spatial Probability Distribution Mapping (PMAP)

**Figure 1:**
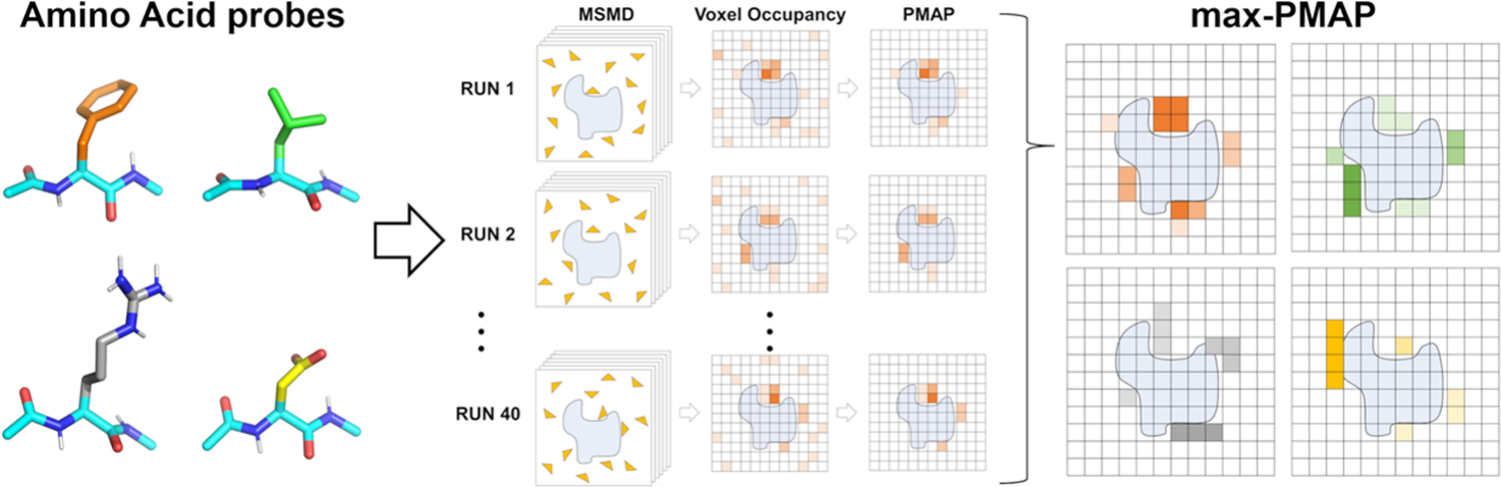
Workflow for max-PMAP construction.

The PPI surface on a protein is favorable for amino acid binding. Therefore, we estimated the PPI surface using the PMAP of the amino acid probes. After the production runs were conducted, all trajectories were processed to generate PMAPs. Side chain heavy atoms of all probes in the snapshots were binned into 1 Å × 1 Å × 1 Å grid voxels, and the voxel occupancy of probe-heavy atoms was counted. Since the PPI surface exists on a protein surface, *V* was a set of voxels within 5 Å from the protein atoms, and the values at voxel *v*∉*V* were discarded to focus on the protein surface. Lastly, each voxel count was converted into occupancy probabilities by dividing them by the number of snapshots. All of the occupancy probabilities in the grid were defined as PMAP. Notably, a PMAP corresponds to a trajectory; therefore, 40 PMAPs were generated for each probe (Figure 1). The largest value among the PMAPs generated from each independent trajectory was stored for each voxel in *V* as the max-PMAP. Even for a deep pocket to which a probe will bind strongly but is difficult to reach, a considerable value of voxel *v* of max-PMAP was observed if the binding occurred at least once.

### Binding Free Energy Prediction Using Grid Free Energy (GFE)

AAp-MSMD can predict the binding affinity by calculating the GFE. Boltzmann-based transformation enables the estimation of the free energy of each position called GFE.^46^ The GFE is defined as
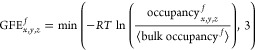
1
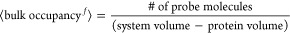
2where *x*, *y*, and *z* correspond to the coordinates of each voxel and *f* corresponds to the probe type. The GFE values were capped at 3 kcal/mol to avoid unphysically high energies. Note that the center of each probe molecule was used to calculate occupancy because utilizing side chain atoms causes high bulk occupancy, narrowing the GFE value range and underestimating binding affinity.

### Target Information

We performed AAp-MSMD for several target proteins that form protein–peptide complexes and assessed the residue-dependent hotspot detection compared with known PPI surfaces of target proteins. Table 1 shows the target proteins, for which residue-dependent hotspot detection was performed, and the target amino acid residues of the peptide. Alanine scanning has identified these residues as key residues for binding to the PPI surface.^47−53^ AAp-MSMD was performed for the hotspot detection of 28 key peptide residues.

**Table 1:** Target Proteins and Amino Acid Residues of Interest for Residue-Dependent Hotspot Detection^a^

protein	PDB ID	key peptide residue	SASA ratio^b^ [%]	AAp-MSMD hotspot detection
AMA1	3ZWZ	Phe2038	0.40	√
		Arg2041	25.45	√
		Met2042	25.07	√
		Pro2044	*99.51*	
ZipA	1F47	Asp7	*73.98*	
		Ile8	4.57	√
		Phe11	9.69	√
		Leu12	19.48	√
XIAP	1G3F	Val2	*57.32*	
		Pro3	*49.82*	
		Ile4	15.22	
MDM2	1YCR	Phe19	1.44	√
		Asp21	*100.00*	
		Leu22	17.58	
		Trp23	4.59	√
MLL	4GQ6	Phe9	2.80	√
		Pro10	4.43	√
		Arg12	18.63	√
		Pro13	4.97	√
HIV integrase	3AVB	Leu2	*99.25*	
		Lys3	*40.74*	√
		Ile4	11.60	√
		Asp5	7.39	√
		Asn6	*40.62*	
uPA	4X1Q	Pro2	7.74	√
		Tyr4	13.81	
		Ser5	*43.38*	
		Arg6	0.66	√

a SASA ratio values above the threshold (30%) are written in italic font.

b SASA ratio: SASA_bound_/SASA_unbound_, where SASA_bound_ and SASA_unbound_ are the solvent-accessible surface areas (SASA) of each key residue for the protein–peptide complex (SASA_bound_) and peptide without target protein (SASA_unbound_). Each SASA was determined by Residue Analysis in Schrodinger suite 2019-4.

**Figure 2:**
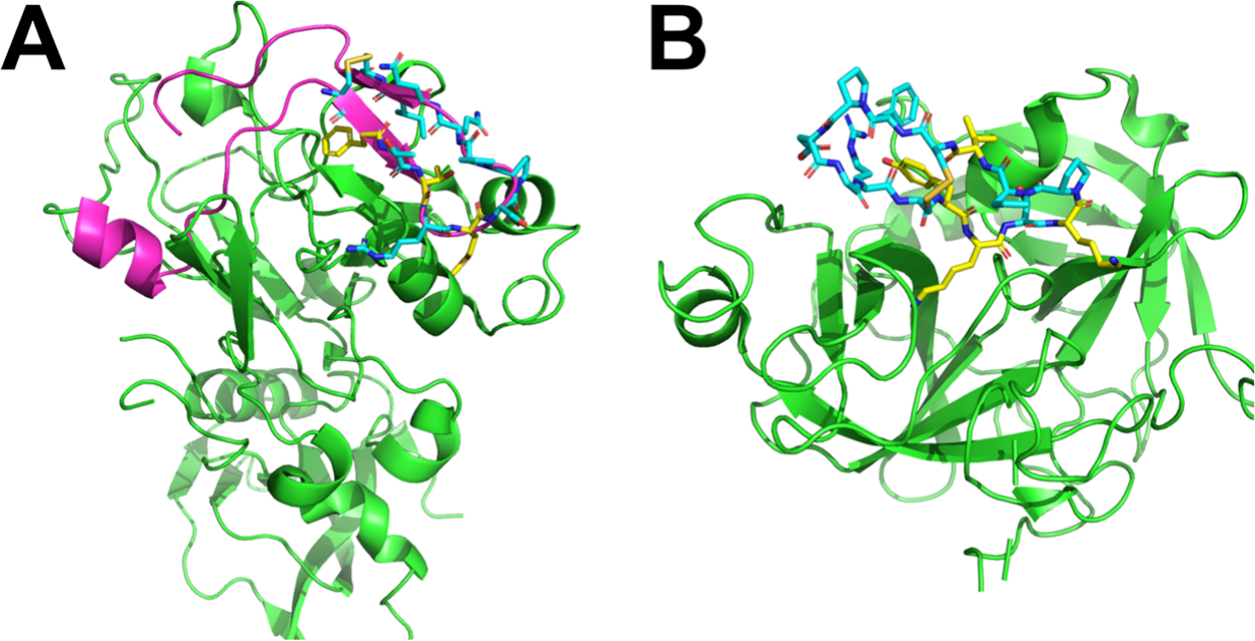
Complex structures of AMA1–RON2sp1 and the plasmin–sunflower trypsin inhibitor. (A) AMA1 protein and RON2sp1 peptide 3D structures are shown in the green and magenta cartoon models, respectively. The stick model indicates that the residues consisted of cyclic peptides. (B) Plasmin protein and cyclic peptide inhibitor 3D structures are shown in the green cartoon and cyan stick models, respectively. The mutated residues with SAR in each peptide are colored yellow.

Next, we selected two target proteins with known peptide inhibitors with SAR data: apical membrane antigen 1 (AMA1) and plasmin. Wang et al. reported the SAR results of the interaction between β-hairpin peptide inhibitor against AMA1 (Figure 2A).^47^ Additionally, Swedberg et al. reported SAR results for sunflower trypsin inhibitor-1 (SFTI-1) against plasmin (Figure 2B).^54^ As for AMA1, we selected 10 peptide analogues with single-point mutations (F2038X, T2040X, and M2042X) and PDB ID 3ZWZ as the initial protein conformation of AAp-MSMD. As for plasmin, we also selected another 5 peptide analogues with single-point mutations (Y4X, K5X, and K7X) and PDB ID 6D3X as the initial protein conformation. In total, 17 AAp-MSMD simulations were conducted (Phe, His, Leu, Met, Gln, Arg, Ser, Thr, Val, Trp, and Tyr probes for AMA1 and Phe, Ile, Lys, Arg, Trp, and Tyr probes for plasmin).

### Preparation of Target Protein

Each protein–peptide 3D complex structure was downloaded from the PDB site, and apo structures were generated to remove the counterpart peptides. Next, apo structures were preprocessed using the Protein Preparation Wizard in Schrodinger suite 2019-4 (Schrodinger, Inc., New York, NY). Missing side chains in each structure were filled using prime. Residues next to the missing loop in the crystal structure were capped with *N*-methyl amide and acetyl capping groups. Afterward, hydrogens of proteins were placed based on the hydrogen bonding and ionization states at pH = 7 using PROPKA.^55^ Asparagine and glutamine residue flips were also handled appropriately. Subsequently, the preprocessed proteins were used as the input structure for the simulation.^35^ For hotspot detection, AAp-MSMD was performed for each target protein–amino acid probe pair, and each max-PMAP was qualitatively compared to each residue position in the peptide. For binding free energy prediction, wild-type (WT) and mutated amino acid types of each SAR residue position were used as probes in the AAp-MSMD. We defined the “residue-GFE” as the best GFE value within 2 Å of the Cα atom and side chain heavy atoms in WT residues of the peptide (Figure S1). Residue-GFE was calculated using the AAp-MSMD trajectory with each amino acid probe types.

### MDpocket for Conventional MD-Based Hotspot Detection

MDpocket is a conventional MD-based hotspot detection method.^56^ This method predicts protein pockets by using a geometric approach. In this study, we performed MDpocket using trajectories obtained from AAp-MSMD with AMA1 and the Arg-probe. The detected frequency map at 10% was compared with max-PMAP at the key residue positions of peptides in protein binding.

## Results

### Hotspot Detection

**Figure 3:**
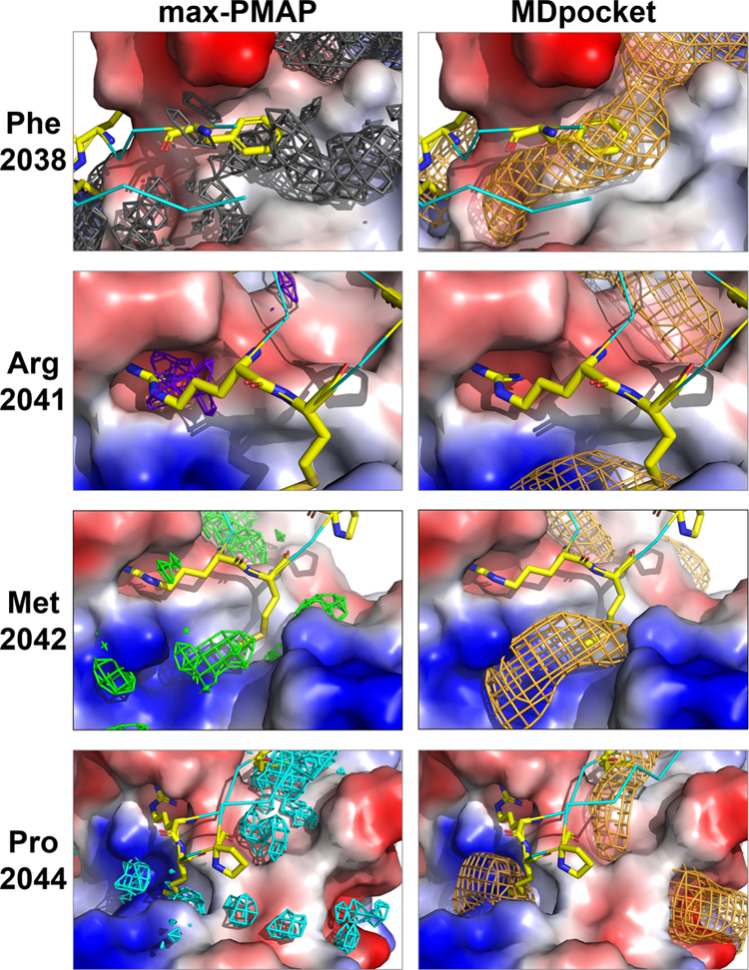
Comparison between max-PMAP of AAp-MSMD and frequency map of MDpocket at each position of the key residue of the β-hairpin peptide in AMA1 binding. The max-PMAPs of Phe, Arg, Met, and Pro-probe are shown as gray, purple, green, and cyan meshes, respectively. Frequency maps of MDpocket are shown as orange meshes. Key residues of cyclic peptide RON2sp1 with a crystal structure are colored yellow. The acidic, basic, and hydrophobic areas in AMA1 are shown as red, blue, and gray surfaces, respectively. The thresholds of max-PMAP were set using the ratio of the number of side-chain heavy atoms of the probes based on visual inspection (Phe:Arg:Met:Pro = 15.0%:15.0%:8.6%:6.4%). The threshold of the frequency map of the MDpocket was set to 10%.

Column 5 in Table 1 shows the results of the residue-dependent hotspot detection. The max-PMAP values at each position in the target proteins are shown in Figures 3 and S2–S7. Numerous max-PMAPs (17/28 cases) could detect each position of the key residue in PPI binding. Hotspot detection for HIV integrase (Figure S6) provides an example of probe dependency, and the position of Lys3 was not detected with the Leu-probe but was detected with the Lys-probe. On the other hand, certain positions in the PPIs could not be detected by AAp-MSMD without probe dependency because these residues in the peptide do not directly interact with the target protein. Figure S2 presents an example and shows that Asp7 was not detected with either the Asp-probe or Ile-probe. To quantitatively assess the peptide interactions with target proteins, we estimated the solvent-accessible surface area (SASA) of each key residue for the protein–peptide complex (SASA_bound_) and the peptide without target protein (SASA_unbound_). We also calculated the ratio of SASA_bound_ to SASA_unbound_ (hereinafter referred to as the SASA ratio). The results showed that most residues that could not be detected by AAp-MSMD were exposed to solvents in the complex structures (Column 4 in Table 1). Excluding the key residues with SASA ratios over 30%, AAp-MSMD had a high detection rate of 84.2% (16/19 cases). These findings indicate that AAp-MSMD can detect key residue binding sites on the target protein surface at the residue-type level.

With a focus on each position of the key residue of cyclic peptide RON2sp1 in AMA1 binding, the max-PMAP of AAp-MSMD with each type of key residue probe and the frequency map of the MDpocket are shown on the left and right sides of Figure 3, respectively. First, AAp-MSMD and MDpocket detected the side chain positions of Phe2038. This binding site is a shallow, wide hydrophobic pocket; therefore, a large area of the periphery was detected by both maps. Furthermore, the side chain positions of Met2042 were detected by both maps because of the deep and wide pockets around the side chain terminus. Hence, AAp-MSMD and MDpocket can easily detect wide pockets, such as those around Phe2038 and Met2042. Notably, MDpocket did not detect the side chain position of Arg2041; however, max-PMAP with an Arg-probe accurately detected this position. This shows that the max-PMAP from AAp-MSMD can detect PPI hotspots more accurately than the conventional method. Additionally, AAp-MSMD and MDpocket could not detect the side chain position of Pro2044. Actually, this residue has less contact with the target protein, and the SASA ratio of Pro2044 is 99.51%. Hence, the position of Pro2044 is not appropriate for AAp-MSMD, as highlighted in the Discussion section.

### Binding Free Energy Prediction for Amino Acid Probe Affinity

**Figure 4:**
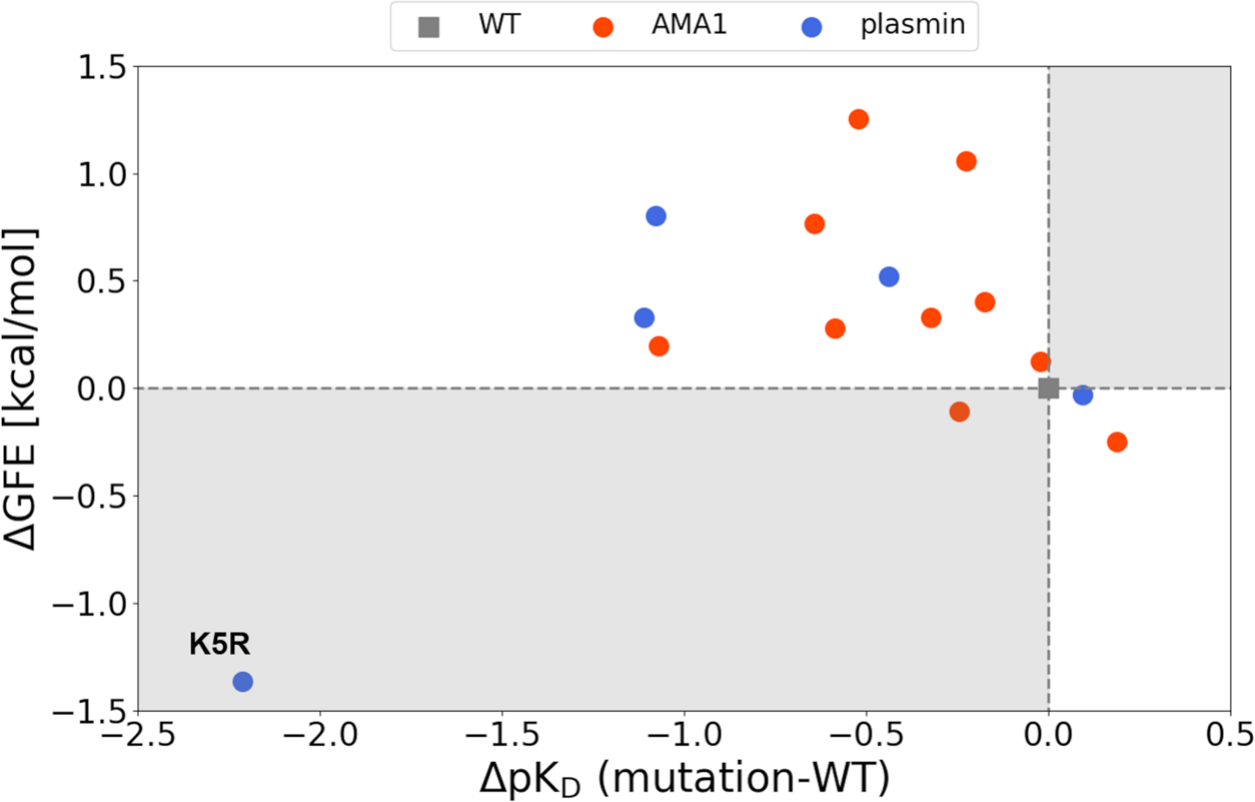
Scatter plot of the experimental binding affinity and ΔGFE. WT residue is represented by a gray dot, and the results of AMA1 and plasmin are represented by orange and blue dots, respectively. The white area indicates that the predicted value is consistent with the binding affinity based on the WT residue.

Figure 4 shows the plot of the energy difference between the WT and mutant. The *x*-axis indicates the experimental binding affinity difference (Δp*K*_D_), and the *y*-axis indicates the residue-GFE energy difference (ΔGFE). These detailed values are shown in the Supporting Information (Table S1). A positive or negative ΔGFE means that the estimated binding affinity of the mutated residue is weaker or stronger than that of the WT residue. The correlation coefficient in these plots was −0.42, excluding the outlier of plasmin (K5R). Furthermore, the figure shows that most ΔGFEs were consistent with a stronger or weaker experimental binding affinity relative to that of the WT residue (14 cases out of 16). It included most analogues, excluding the value of T2040F in AMA1 (Figure S8), and Y4X and K7X in plasmin (Figure S9). Notably, these ΔGFEs tended to match the experimental values despite the slight binding affinity differences between the WT and mutated residues such as M2042F in AMA1 and K7R in plasmin (Table S1). These results suggest that AAp-MSMD can be used to roughly estimate the experimental binding affinity even if the range of the values is less than two. However, the K5R in plasmin result showed that ΔGFE was overestimated compared to the experimental binding affinity. This case is revisited in the Discussion section.

## Discussion

### Examination for the Probe Structure in the AAp-MSMD

**Figure 5:**
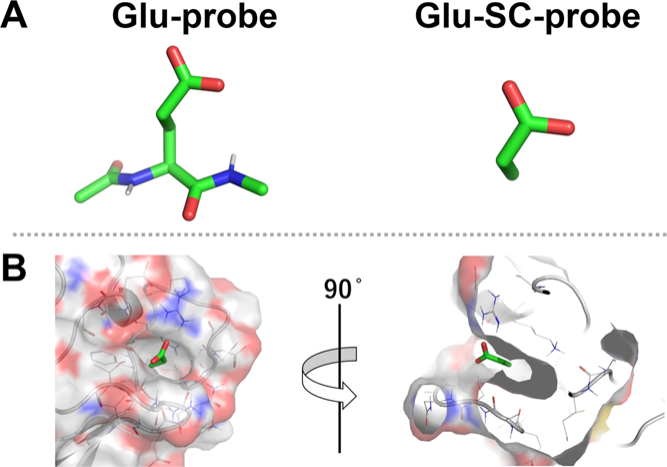
Examination of a probe structure of glutamic acid. (A) Comparison of chemical structures between the Glu-probe and Glu-SC-probe. (B) Example for the binding mode of the Glu-SC-probe in AMA1. The gray lines, cartoon, and surface represent the target protein, and the green sticks represent the predominant binding state of the Glu-SC-probe.

Conventional MSMD uses rigid probes because probes with high flexibility cause sampling inefficiency. MSMD using amino acid side chain probes, which are less flexible than amino acid probes with main chain atoms, may have efficient sampling and reduction in the cost of computing. Therefore, MSMD was performed using an amino acid side chain probe (SC-probe, Figure 5A) to validate the original amino acid probe. The SC-probe was modeled by deleting the backbone atoms from each 3D structure. The structure and electrostatic potential of the SC-probes were optimized by using the same procedure applied for the original probes. Furthermore, MSMD was performed under the same conditions to produce the max-PMAPs.

In this section, the cosolvent structure of glutamic acid is discussed (Supporting Information includes a comparison with the SC-probe of several amino acid types). Figure 5B shows an example of the binding state of the Glu-SC-probe in AMA1. In the binding mode, the Cβ atom in the Glu-SC-probe was inside the deep pocket. However, the binding site was too small and deep to place the backbone atoms based on the binding mode of the Glu-SC-probe. This indicates that glutamic acid in the peptides does not have this binding mode. This misdetection of the binding site was derived from the out control of the direction of the SC-probe. However, the Glu-probe, which contains main chain atoms, cannot have this binding state because these main chain atoms block the entry of the binding site in the same direction. This suggests that the original amino acid probe can prevent such misdetection, because of the directional control provided by the backbone atoms in the probe. Therefore, the max-PMAP of the SC-probe without main chain atoms is inappropriate for detecting the PPI surface to which the amino acid binds. In contrast, the original amino acid probe with main chain atoms is appropriate for valid hotspot detection and accurate binding free energy prediction from MSMD calculations.

### Residue-Dependent Hotspot Detection

**Figure 6:**
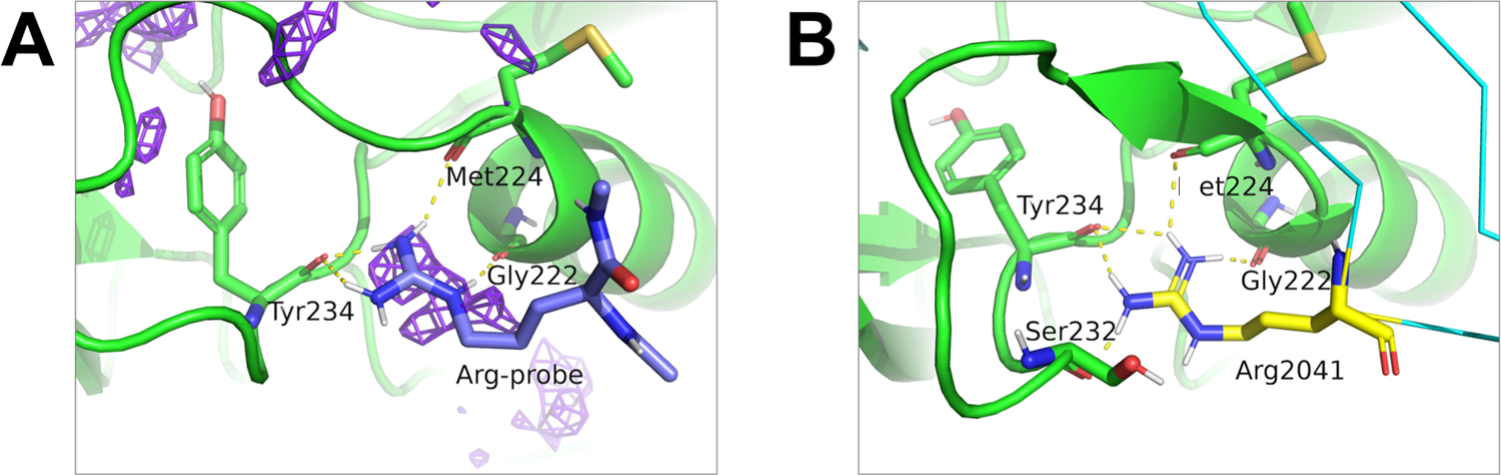
Binding state of Arg-probe and Arg2041 in RON2sp1. (A) Predominant binding state of the Arg-probe of AAp-MSMD trajectories. (B) Binding state of Arg2041 in the AMA1-RON2sp1 complex structure. AMA1, RON2sp1, Arg2041, and Arg-probe are colored green, cyan, yellow, and purple, respectively. Max-PMAP of the Arg-probe is shown as a purple mesh. Polar contact between the side chain of arginine and AMA1 is shown as dash lines.

In hotspot detection, the detection of the binding site at Arg2041 is the characteristic point of max-PMAP. This acidic binding site is too small to bind common small molecules and bulky amino acid residues; however, it is accessible for arginine. Notably, hotspot detection of max-PMAP using AAp-MSMD could accurately detect these selective binding sites. The binding poses of the Arg-probe were similar to those of Arg2041 in the AMA1-RON2sp1 protein–peptide complex (Figure 6). During Arg-probe binding, the side chain atoms in the probe interact with the backbone atoms of Gly222, Met224, and Tyr234 in AMA1 (Figure 6A). Similarly, Arg2041 interacts with the backbone atoms of Gly222, Met224, Ser232, and Tyr234 in AMA1 (Figure 6B). These results suggest that AAp-MSMD can search for PPI sites at the all-atom interaction level.

### Residues Far from the Protein Surface

**Figure 7:**
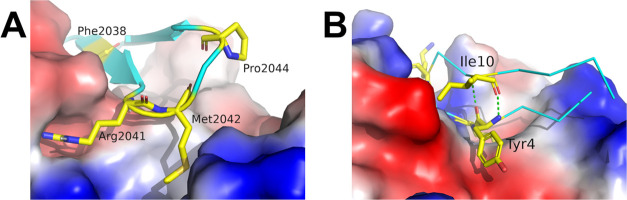
Binding state of the protein–peptide complex structures. (A) AMA1 and RON2sp1 complex structure. (B) Plasmin and SFTI-1 complex structure. Target proteins are shown as a surface model. Peptides and key residues are colored cyan and yellow, respectively. Intramolecular hydrogen bonds are shown as green dashed lines.

In residue-dependent hotspot detection for key residues of cyclic peptide RON2sp1 with AMA1 binding, both AAp-MSMD and MDpocket failed to detect the side chain positions of Pro2044 because Pro2044 in the peptide-bound state is far from the protein surface. Notably, Pro2044 is the key residue that stabilizes the β-hairpin peptide conformation rather than the interaction between AMA1 and Pro2044 (Figure 7A). AAp-MSMD detects hotspots based on protein–probe interactions and is not subject to the residue position that contributes to the stable conformation of the peptide. Therefore, the failure of both methods to detect the position of Pro2044 is not a significant problem.

In the binding free energy prediction of plasmin, we also performed AAp-MSMD for the residues of I10X to further investigate the abovementioned issue. Ile10 is stable with hydrogen bonds between the main chain atoms in Ile10 and Tyr4 (Figure 7B), and it hardly interacts with the protein surface, which is similar to Pro2044 of the RON2sp1 peptide. The SASA ratio of Ile10 is 39.88%, which is above the threshold. According to the SAR results, Ile, which is a WT residue, has the highest experimental affinity compared to that of the mutations; however, residue-GFE in I10X has the lowest estimated affinity among these residues (Table S2). Since AAp-MSMD uses the amino acid monomer probes, a stable pose, such as that of Ile10 in the complex structure, cannot be seen in the simulation. This result indicates that the threshold of 30% for the SASA ratio is still effective, even for binding free energy prediction using AAp-MSMD.

### Probe Orientation Control on the Open and Shallow Protein Surface

AAp-MSMD can be used to observe various probe-binding states in the target protein surface because the probe does not have orientation constraints in the simulation. Notably, these binding states in the open and shallow pockets of the protein are diverse. This aspect of the probe in AAp-MSMD is appropriate for hotspot detection, which exhaustively searches for the protein surface by considering diverse binding states.

**Figure 8:**
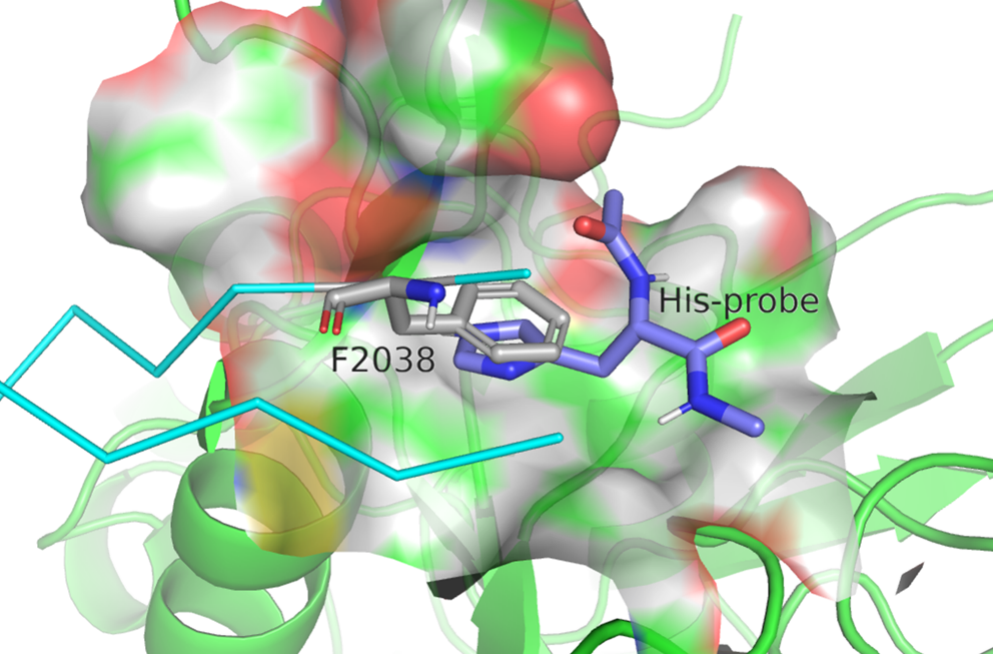
Example binding state of the His-probe at the position of Phe2038. AMA1, β-hairpin peptide, and Phe2038 are shown as green cartoons, cyan ribbons, and gray stick models, respectively. Purple sticks represent the binding states of His-probes. The binding state was selected by visual inspection of AAp-MSMD trajectories.

Furthermore, an energy-based evaluation of the probe in AAp-MSMD should be noted. Originally, amino acid residues in peptides are constrained by their backbone, immobilizing their binding orientations; therefore, the GFE should be calculated under the constraints of the probe orientation based on the position of the peptide backbone. However, this issue seldom occurs for binding in the small and narrow pockets because the probe orientations are constrained by their binding sites. Energy-based evaluation at the positions of T2040X and M2042X revealed that their ΔGFE was moderately consistent with the experimental binding affinities obtained from SAR. In contrast, in the open and shallow pockets, such as the position of F2038X, ΔGFE was overestimated caused by the out control of the probe conformation (Figure 8) and the combination of probe occupancy derived from diverse orientations. Our GFE represents the probe’s center-based GFE; however, this overestimation can be overcome by generating atom-based GFEs,^57^ which implicitly have an orientation preference of the probe. If atom-based GFEs are applied, then all atoms in the probe will be distinguished, even if they are the same element, and GFEs will be generated per atom. Atom-based GFEs show the preferred positions of the main chain Cα atom and the side chain carbon atoms separately, indicating the preferred orientations in a binding site. This extension should be the focus of future studies.

### Improvement of GFE by Adequate Sampling

According to the SAR results and our binding free energy prediction of SFTI-1 against plasmin, the results for the ΔGFE of K5R were opposite to those of the experimental Δp*K*_D_. This discrepancy was caused by the excessively low residue-GFE of the Lys-probe. The deep binding site in plasmin may have induced insufficient sampling, resulting in the low residue-GFE. Hence, we performed additional sampling of AAp-MSMD using the Lys-probe and recalculated residue-GFE by increasing the number of runs of MSMD sampling from 40 to 80. As a result, residue-GFE of the Lys-probe and ΔGFE of K5R improved to −4.422 and 0.038 kcal/mol, respectively (Table S3). This case can occur when the amino acid probe has high flexibility, such as with the Lys-probe. Additional runs will improve the accuracy of residue-dependent hotspot detection and binding free energy prediction. However, the computational costs are high (e.g., 3.2 μs for a protein–probe pair with 80 runs of 40 ns simulations). The required sampling depends on the protein–probe pair of interest; thus, the total MSMD simulation length should be adjusted. A possible option is the use of an overlap coefficient (OC)^58^ between a max-PMAP of N runs and a max-PMAP of N-1 runs. The sampling may be sufficient if the coefficient is above a threshold. Furthermore, Smith and Carlson conducted MSMD using accelerated MD (aMD) to enhance sampling efficiency.^59^ Combining AAp-MSMD with aMD may contribute to adequate sampling.

## Conclusions

We proposed AAp-MSMD, a mixed-solvent molecular dynamics simulation using amino acid probes that enables hotspot detection of PPI sites and amino acid binding free energy prediction at a PPI site. In hotspot detection, 84.2% of the binding sites of the PPIs showing contact with the target protein could be detected at the residue-type level. The max-PMAP obtained from AAp-MSMD detected the PPI site of AMA1 more accurately than the conventional pocket detection method, MDpocket. Furthermore, in the binding free energy prediction using an amino acid probe, ΔGFE obtained from AAp-MSMD moderately estimated the experimental binding affinities of most peptide analogues. In particular, AAp-MSMD is effective for identifying residue positions in contact with the target protein. In addition, unlike SC-probes without a main chain, our amino acid probes with main chains controlled the direction of probe binding by the main chain atoms and detected peptide-accessible hotspots by universally searching for diverse probe-binding orientations. This universal search strategy is effective for small and narrow pockets. However, the universal orientation of the probe-binding state sometimes caused an overestimation of the residue-GFE. Nevertheless, this overestimation can be overcome by generating atom-based GFEs, which implicitly have an orientation preference of the probe.

## Data Availability

Initial 3D structures of the protein, peptides, and probes were downloaded from the Protein Data Bank (PDB). Schrodinger suite 2019-4 was used for protein preparation and SASA calculation. We used AmberTools21 and Gaussian 16 Rev B.01 for probes preparation. PACKMOL 18.169 and AmberTools18 were used to prepare the MSMD system. GROMACS 2021.5 was used as the MD engine. PyMOL was used for visualization. The protocol of MSMD simulation in this study is available from the following link: https://github.com/keisuke-yanagisawa/exprorer_msmd. MDpockets were used to compare the hotspot detection methods. The input data (all protein structures, all probe structures, and ten AAp-MSMD initial systems per protein–probe pair), ten 40 ns trajectories consisting of snapshots every 500 ps per protein–probe pair, and GFEs in OpenDX format (.dx) are deposited to Zenodo (DOI: 10.5281/zenodo.10016348).

## Supporting Information Available

Region for calculating residue-GFE (Figure S1); residue-dependent hotspot detection for each target protein (Figures S2–S7); scatter plots of the experimental binding affinity and ΔGFE in AMA1 and plasmin (Figures S8 and S9); binding affinity from the SAR and GFE from AAp-MSMD (Table S1); examples of protein–probe binding structures in the amino acid probe MSMD (Figures S10–S12); binding affinity from I10X in plasmin and GFE from AAp-MSMD (Table S2); and changes in the estimated binding affinity of the Lys5 position in plasmin with a different number of replicas (Table S3) (PDF)

## Abbreviations

AAp-MSMD: amino acid probe-based mixed-solvent molecular dynamics
AMA1: apical membrane antigen 1
GAFF2: general AMBER force field 2
GFE: grid free energy
HIV: human immunodeficiency virus
MDM2: murine double minute 2
MLL: mixed lineage leukemia
MSMD: mixed-solvent molecular dynamics
OC: overlap coefficient
PDB: Protein Data Bank
PMAP: spatial probability distribution map
PPI: protein–protein interaction
RESP: restrained electrostatic potential procedure
RON2: rhoptry neck protein 2
SAR: structure–activity relationship
SFTI-1: sunflower trypsin inhibitor-1
uPA: urokinase-type plasminogen Activator
WT: wild type
XIAP: X-linked inhibitor-of-apoptosis protein
ZipA: Z interacting protein A
