# Supplementary material for: AAp-MSMD: Amino Acid Preference Mapping on Protein–Protein Interaction Surfaces Using Mixed-Solvent Molecular Dynamics
Source: J Chem Inf Model. 2023 Dec 12;63(24):7768–77. doi: 10.1021/acs.jcim.3c01677 (PMC10751795; doi:10.1021/acs.jcim.3c01677)
Supplement: Supplementary file 1 — ci3c01677_si_001.pdf [file ci3c01677_si_001.pdf]

Supporting Information for:

AAp-MSMD: Amino Acid Preference Mapping on Protein-Protein Interaction Surface Using Mixed-Solvent Molecular Dynamics

Genki Kudo[1†], *Keisuke Yanagisawa*[2,3†*], *Ryunosuke Yoshino*[4,5*], *Takatsugu Hirokawa*[4,5]

[1] Physics Department, Graduate School of Pure and Applied Sciences, University of Tsukuba, 1-1-1 Tennodai, Tsukuba 305-8571, Ibaraki, Japan.

[2] Department of Computer Science, School of Computing, Tokyo Institute of Technology, 2-12-1 Ookayama, Meguro 152-8550, Tokyo, Japan.

[3] Middle Molecule IT-based Drug Discovery Laboratory, Tokyo Institute of Technology, 2-12-1 Ookayama, Meguro 152-8550, Tokyo Japan.

[4] Faculty of Medicine, University of Tsukuba, 1-1-1 Tennodai, Tsukuba 305-8575, Ibaraki, Japan.

[5] Transborder Medical Research Center, University of Tsukuba, 1-1-1 Tennodai, Tsukuba 305-8577, Ibaraki, Japan.

[*] To whom correspondence should be addressed;

[†] The authors wish it to be known that, in their opinion, the first two authors should be regarded as joint First Authors.

Email: K.Y.: yanagisawa@c.titech.ac.jp; R.Y.: yoshino.r.aa@md.tsukuba.ac.jp

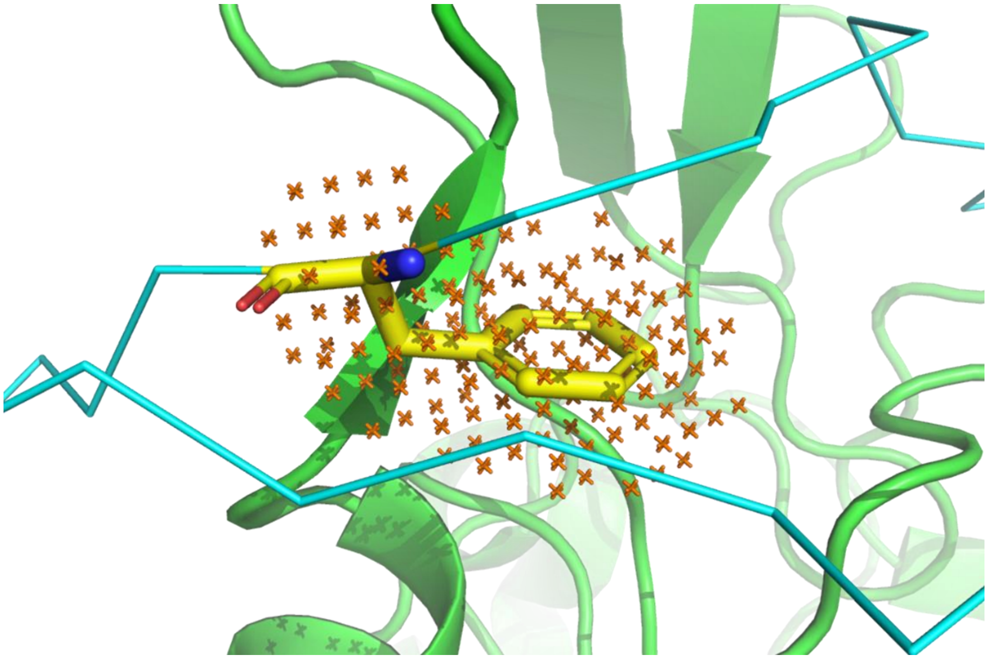

Figure S1. Region used to calculate residue-GFE. Protein, peptide with crystal structure, residue of interest, and region are illustrated as the green cartoon, cyan stick, yellow stick, and brown dots, respectively.

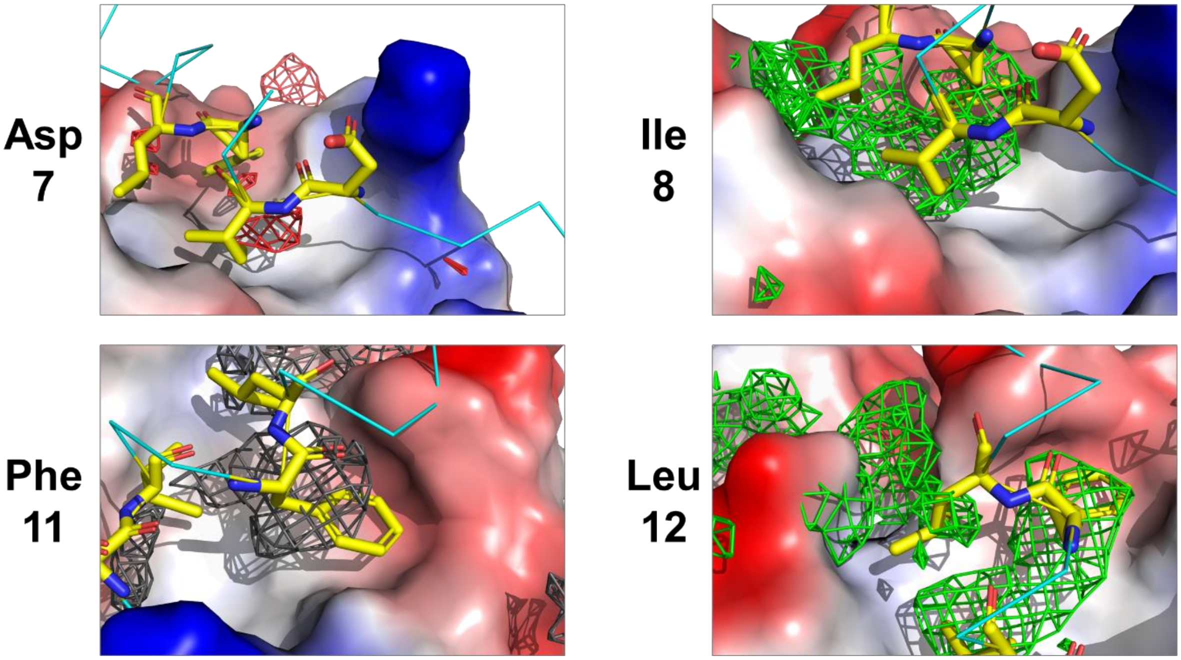

Figure S2. Residue-dependent hotspot detection for ZipA. Target protein is shown as an electrostatic surface, key residues in peptide are shown as yellow sticks, and Max-PMAP of each residue type is shown as a mesh model.

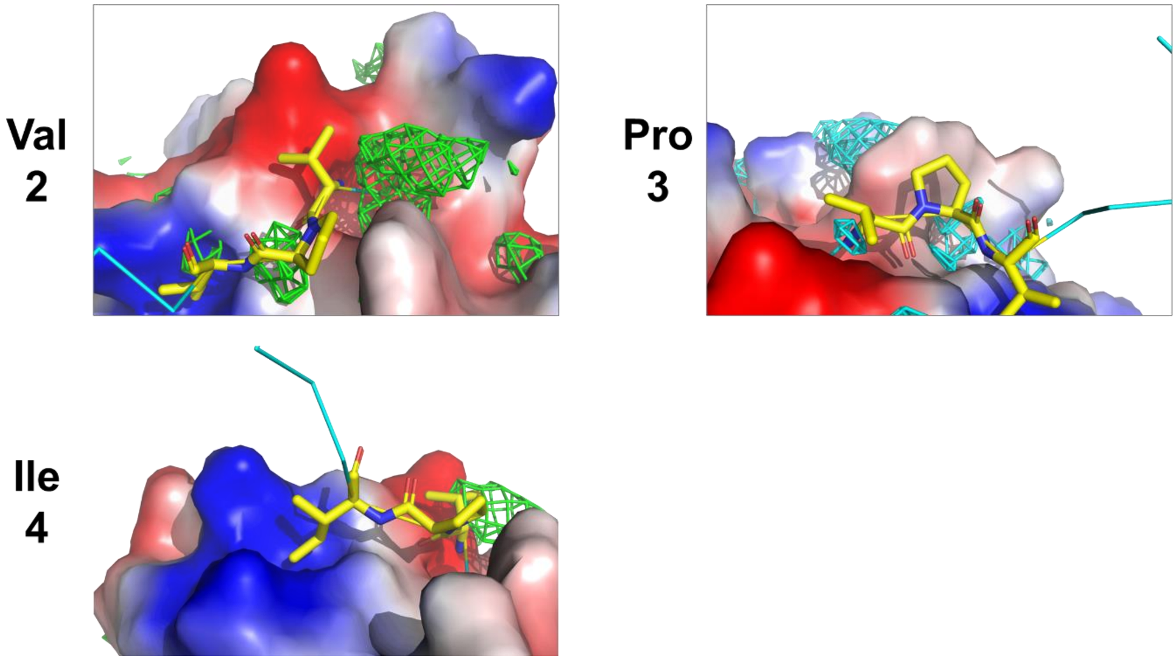

Figure S3. Residue-dependent hotspot detection for XIAP. Target protein is shown as an electrostatic surface, key residues in peptide are shown as yellow sticks, and Max-PMAP of each residue type is shown as a mesh model.

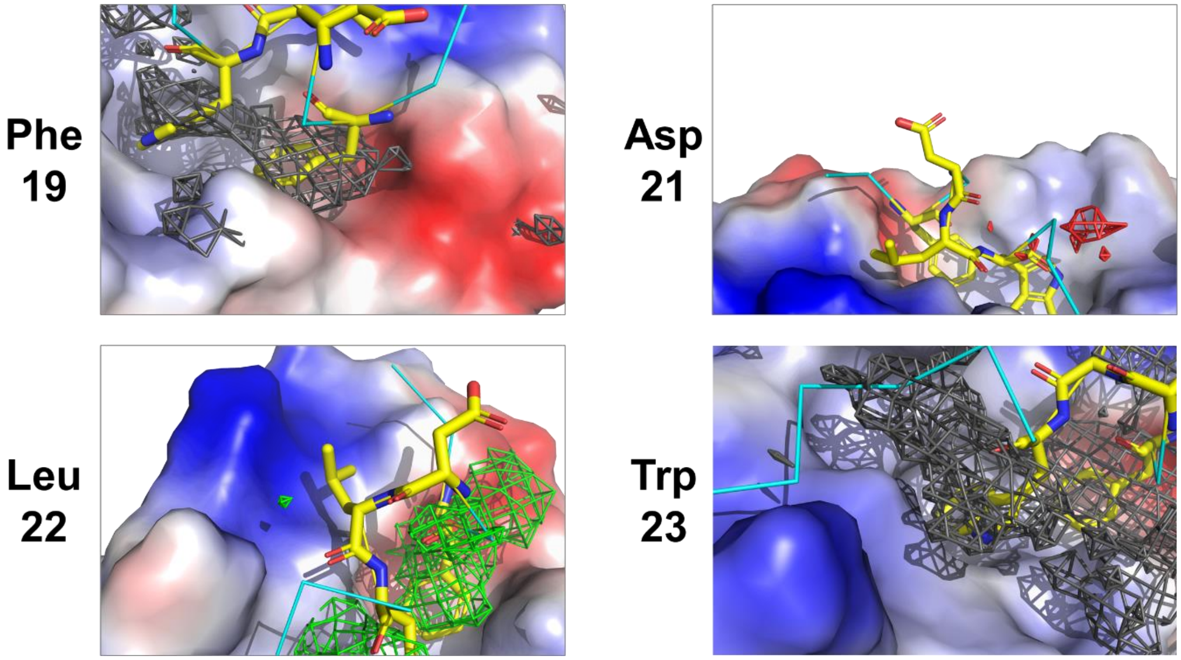

Figure S4. Residue-dependent hotspot detection for MDM2. Target protein is shown as an electrostatic surface, key residues in peptide are shown as yellow sticks, and Max-PMAP of each residue type is shown as a mesh model.

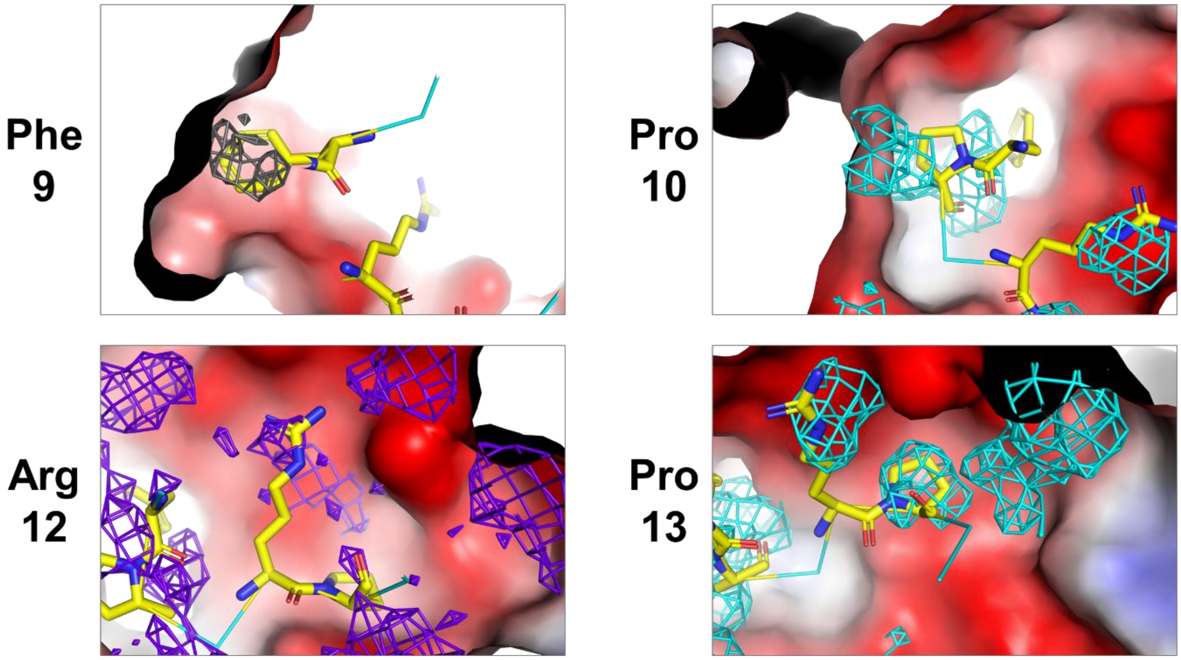

Figure S5. Residue-dependent hotspot detection for MLL. Target protein is shown as an electrostatic surface, key residues in peptide are shown as yellow sticks, and Max-PMAP of each residue type is shown as a mesh model.

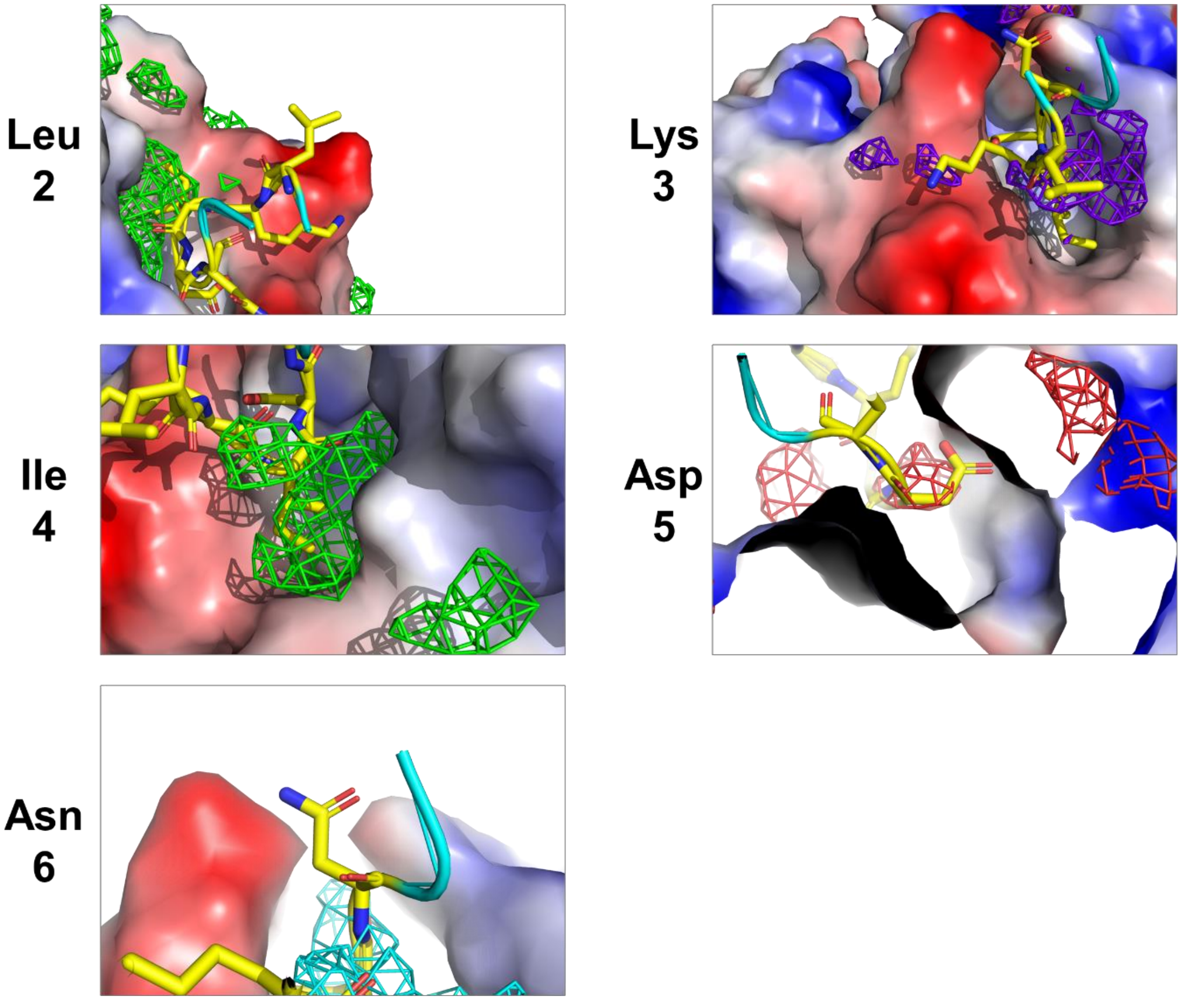

Figure S6. Residue-dependent hotspot detection for HIV integrase. Target protein is shown as an electrostatic surface, key residues in peptide are shown as yellow sticks, and Max-PMAP of each residue type is shown as a mesh model.

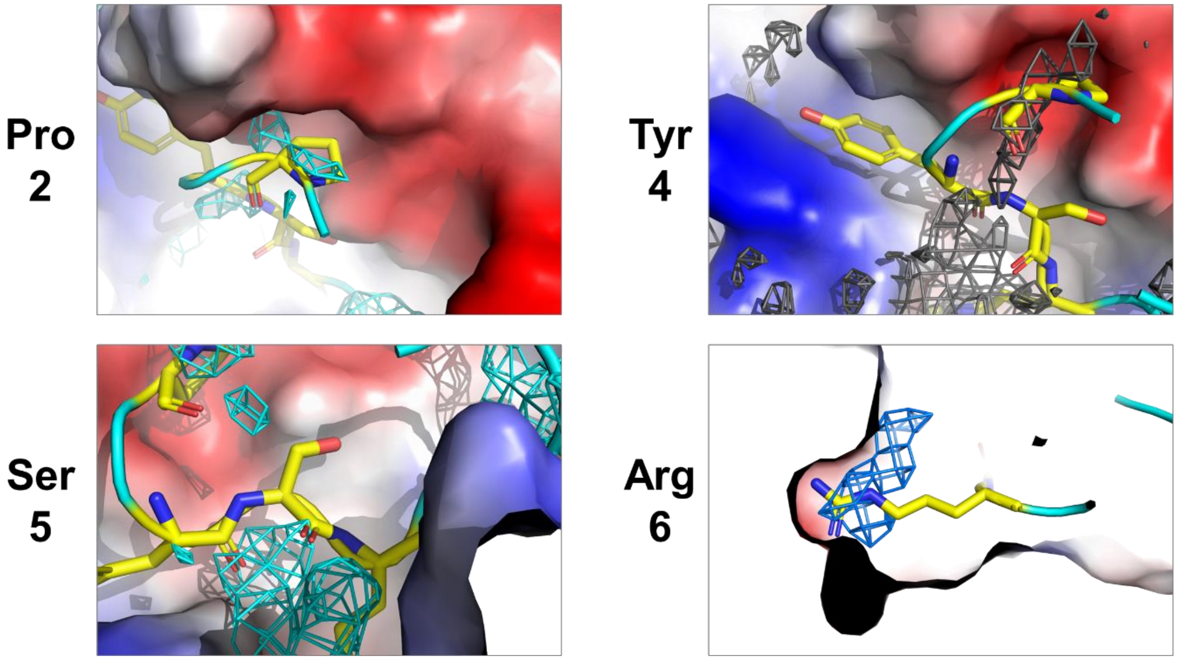

Figure S7. Residue-dependent hotspot detection for uPA. Target protein is shown as an electrostatic surface, key residues in peptide are shown as yellow sticks, and Max-PMAP of each residue type is shown as a mesh model.

Table S1. Binding affinity from the structure-activity relationship and GFE from AAp-MSMD

	Peptides	$-\log K_D$	$\Delta\mathrm{pK_D}$	Residue-GFE (kcal/mol)	ΔGFE from WT (kcal/mol)
AMA1 (3ZWZ)	WT (F2038)	4.244	(0.000)	−4.475	(0.000)
	F2038W	4.432	0.188	−4.722	−0.247
	F2038Y	3.921	−0.323	−4.148	0.327
	F2038H	3.174	−1.070	−4.279	0.196
	WT(T2040)	4.222	(0.000)	−4.430	(0.000)
	T2040S	3.721	−0.523	−3.175	1.255
	T2040V	3.658	−0.587	−4.149	0.281
	T2040F	4.000	−0.244	−4.539	−0.109
	WT(M2042)	4.222	(0.000)	−4.636	(0.000)
	M2042R	4.018	−0.226	−3.578	1.058
	M2042Q	3.602	−0.642	−3.872	0.764
	M2042L	4.071	−0.174	−4.232	0.404
	M2042F	4.222	−0.022	−4.510	0.126

Plasmin (6D3X)					
	WT(Y4)	10.292	(0.000)	−4.953	(0.000)
	Y4F	9.215	−1.078	−4.149	0.804
	Y4W	9.180	−1.112	−4.624	0.329
	WT(K5)	10.292	(0.000)	−3.022	(0.000)
	K5R	8.081	−2.212	−4.384	−1.362
	WT(K7)	10.292	(0.000)	−4.407	(0.000)
	K7I	9.854	−0.439	−3.885	0.522
	K7R	10.387	0.095	−4.437	−0.030

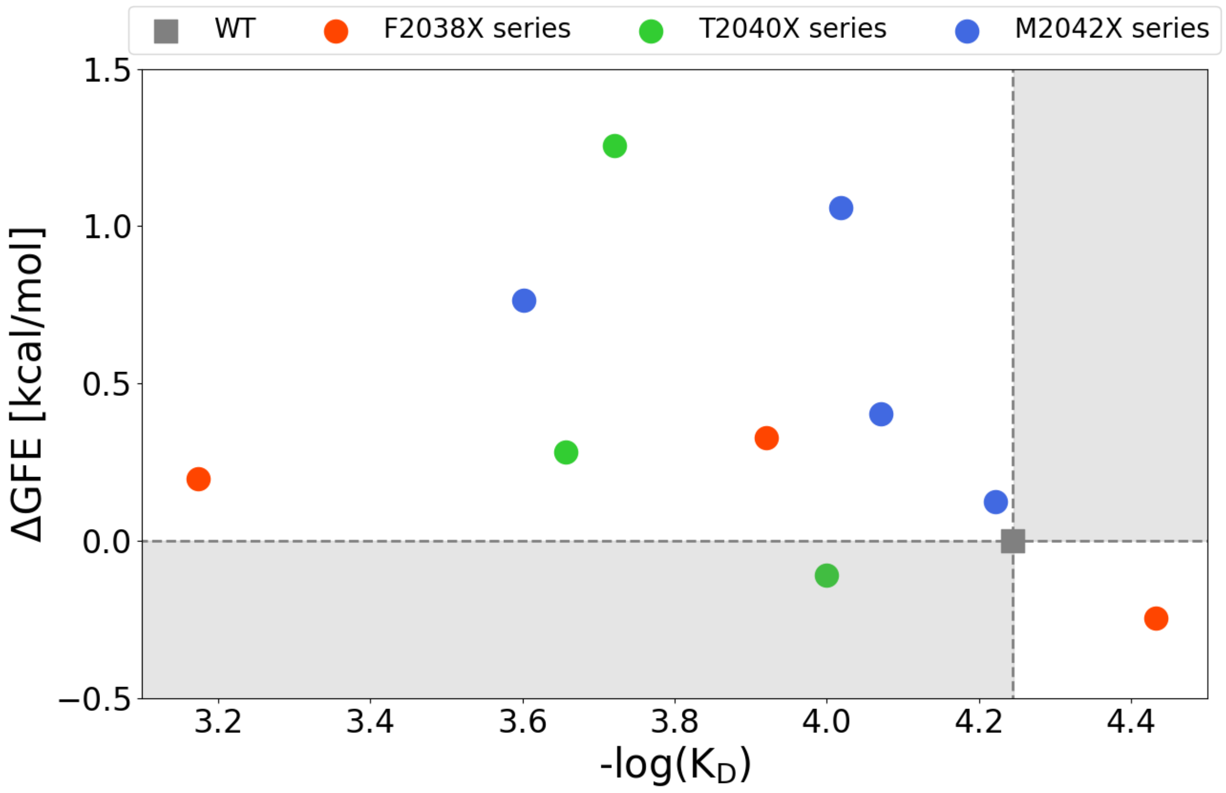

Figure S8. Scatter plot of the experimental binding affinity and ΔGFE in AMA1. WT residue is represented by a gray dot, and F2038X, T2040X, and M2042X are represented by orange, green, and blue dots, respectively. The white area indicates that the predicted value is consistent with the binding affinity based on the WT residue.

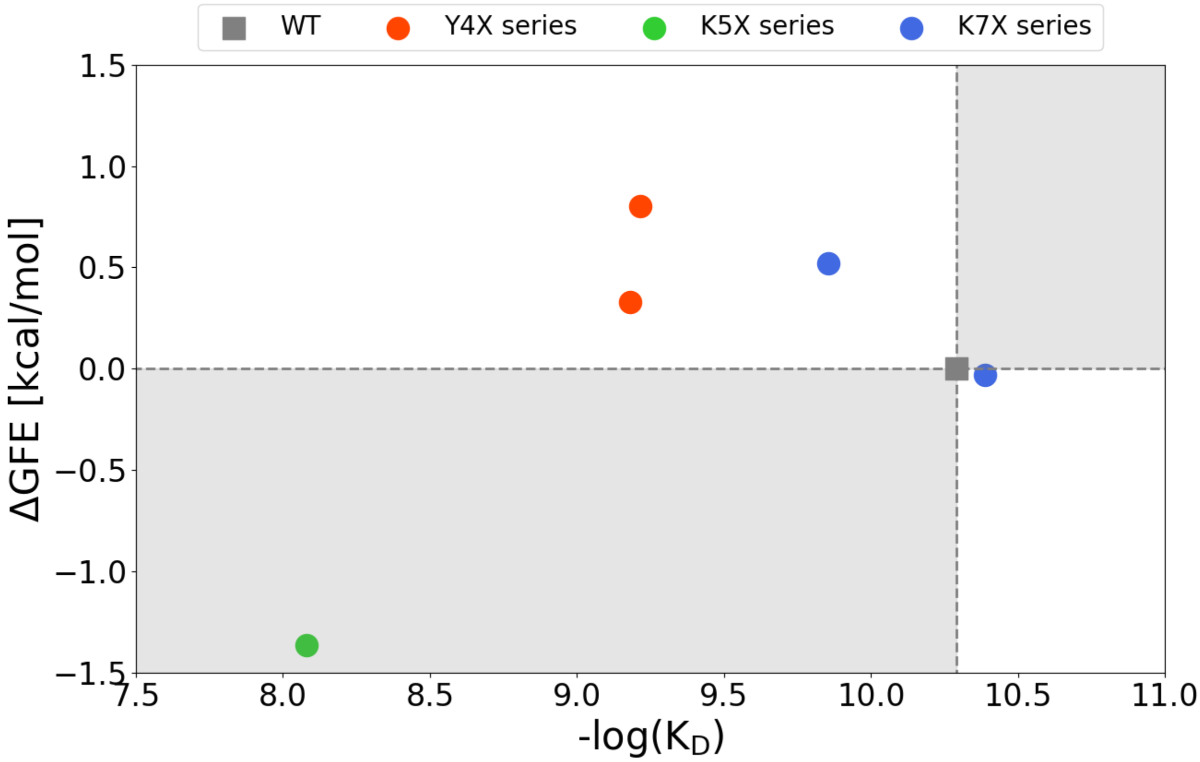

Figure S9. Scatter plot of the experimental binding affinity and ΔGFE in plasmin. WT residue is represented by a gray dot, and Y4X, K5X, and K7X are represented by orange, green, and blue dots, respectively. The white area indicates that the predicted value is consistent with the binding affinity based on the WT residue.

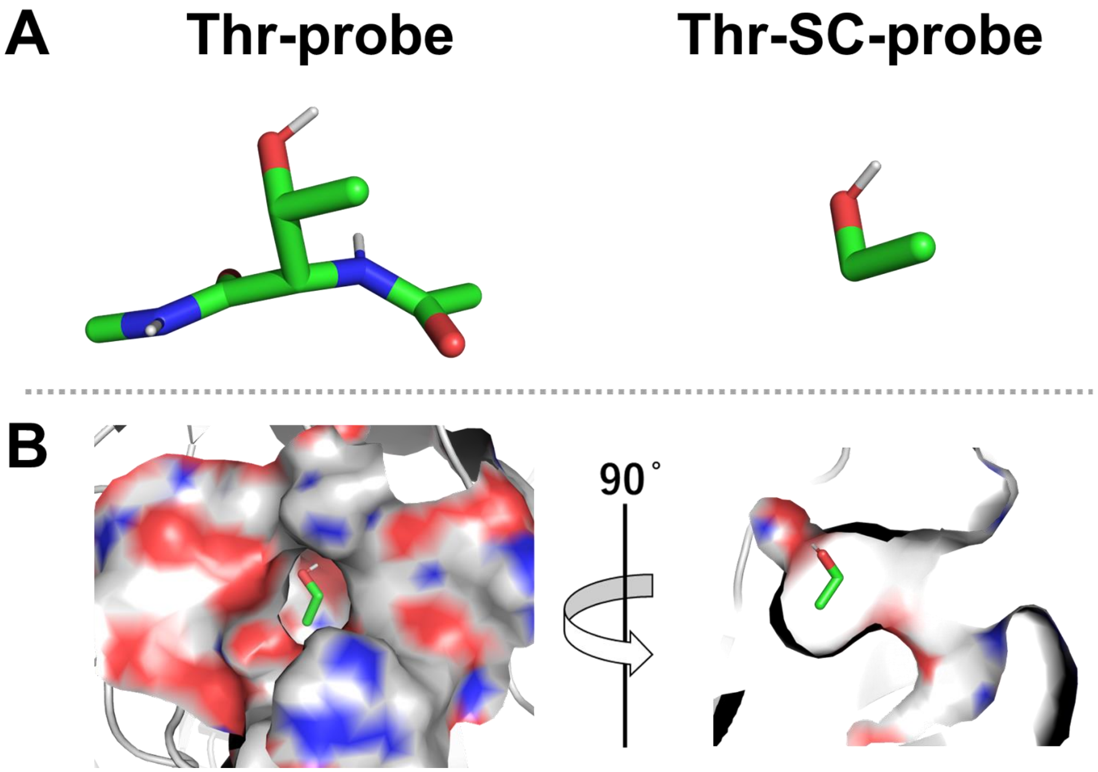

Figure S10. Examination of threonine probe structure. (A) Comparison of chemical structures of Thr-probe and Thr-SC-probe. (B) Example of the binding mode of the Thr-SC-probe to AMA1. Gray lines, cartoons, and surfaces represent the target protein, and green sticks indicate the predominant binding state of the Thr-SC-probe.

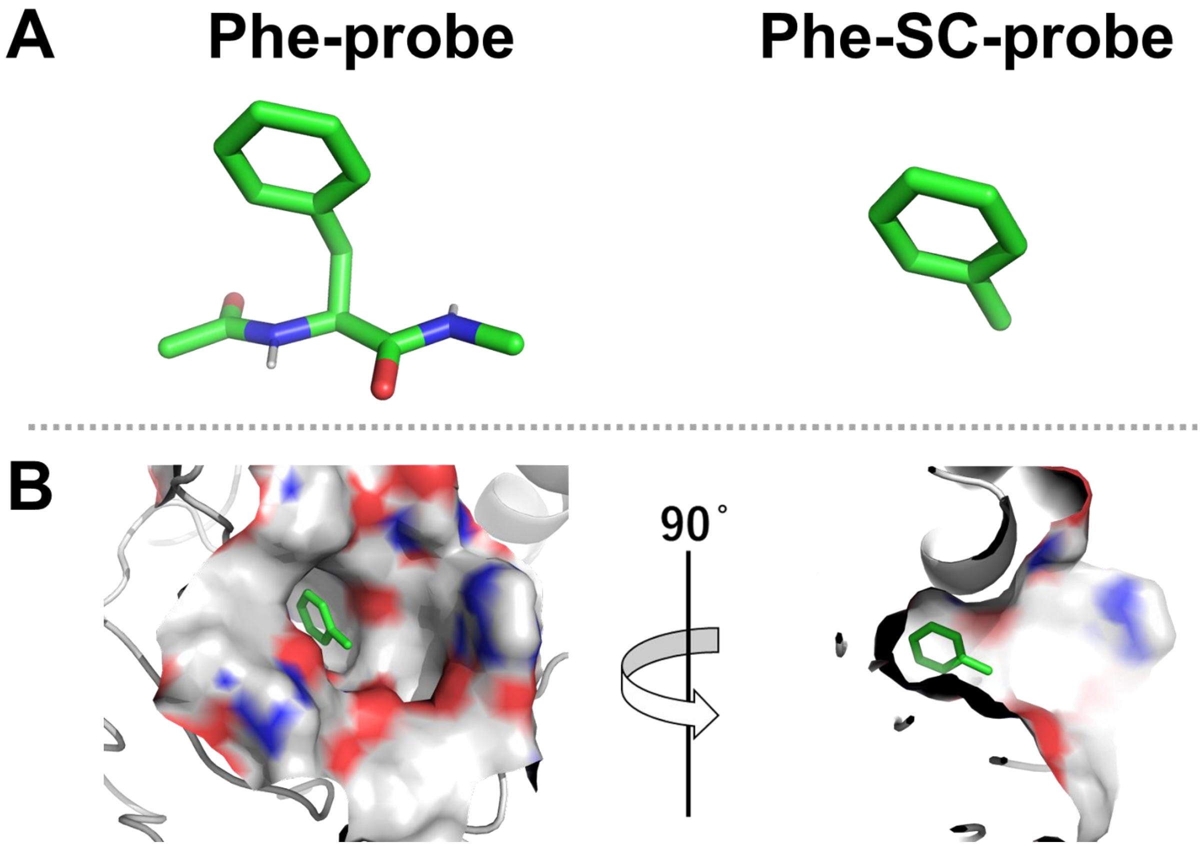

Figure S11. Examination of phenylalanine probe structure (A) Comparison of the chemical structures of the Phe-probe and Phe-SC-probe. (B) Example of the binding mode of the Phe-SC-probe to AMA1. Gray lines, cartoons, and surfaces represent the target protein, and green sticks indicate the predominant binding state of the Phe-SC-probe.

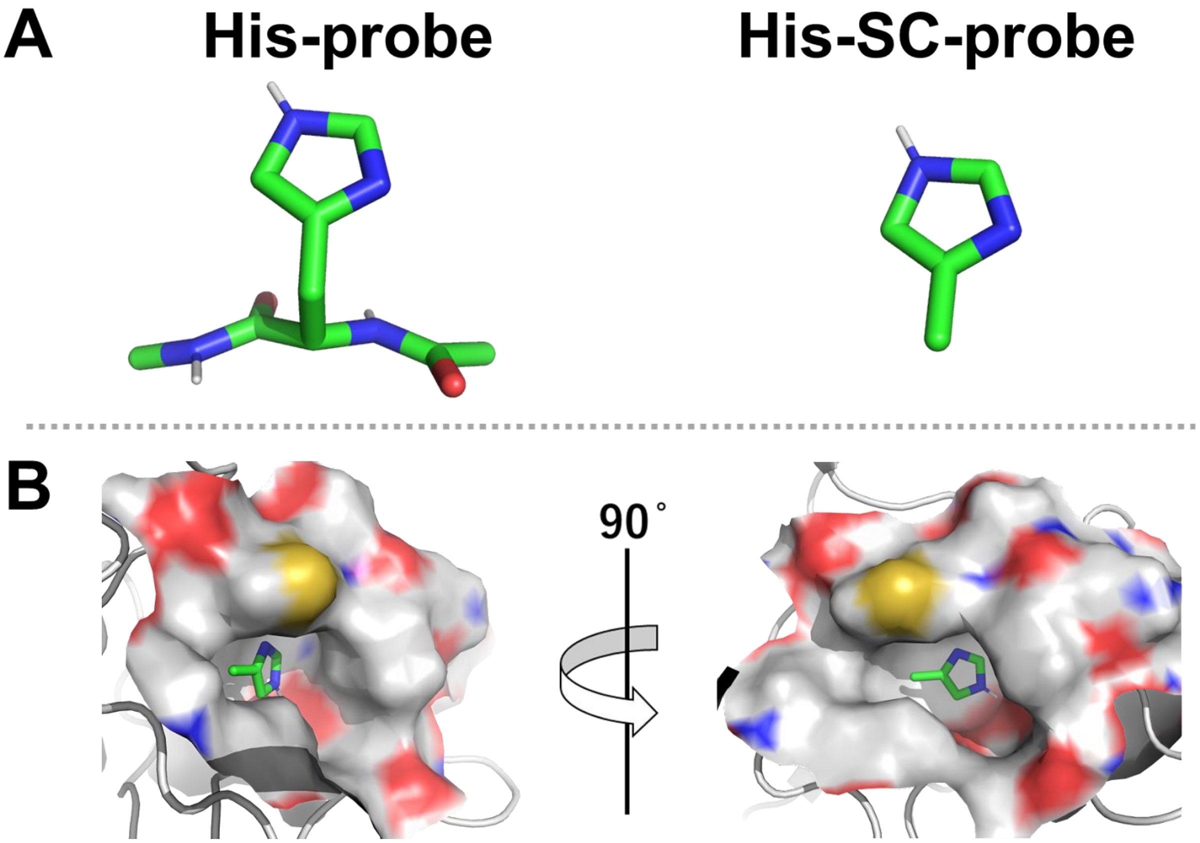

Figure S12. Examination of histidine probe structure. (A) Comparison of the chemical structures of His-probes and His-SC-probes. (B) Example of the binding mode of the His-SC-probe to AMA1. Gray lines, cartoons, and surfaces represent the target protein, and green sticks indicate the predominant binding state of the His-SC-probe.

Table S2. Binding affinity of I10X in plasmin and GFE from AAp-MSMD

Peptides	$-\log K_D$	$\Delta \mathrm{pK_D}$	Residue-GFE (kcal/mol)	ΔGFE from WT (kcal/mol)
WT(I10)	10.292	(0.000)	−2.223	(0.000)
I10K	8.602	−1.690	−3.260	−1.037
I10R	8.328	−1.965	−2.250	−0.027
I10Q	8.745	−1.548	−3.264	−1.041
I10N	9.180	−1.112	−3.184	−0.961

Table S3. Changes in estimated binding affinity of Lys5 position in plasmin with diffirent numbers of replicas.

Peptides	$-\log K_D$	$\Delta \mathrm{pK_D}$	Residue-GFE (kcal/mol)	ΔGFE from WT (kcal/mol)
WT (40 runs)	10.292	(0.000)	−3.022	(0.000)
K5R (40 runs)	8.081	−2.212	−4.384	−1.362
WT (80 runs)	10.292	(0.000)	**−4.422**	(0.000)
K5R (80 runs)	8.081	−2.212	−4.384	**0.037**
